# A case report of autoimmune GFAP astrocytopathy presenting with abnormal heart rate variability and blood pressure variability

**DOI:** 10.1186/s12883-023-03070-4

**Published:** 2023-01-17

**Authors:** Pu-yuan Wen, Guang-qiang Wang, Lian-wei Dou, Qi Chen, Xian-wen Chen, Li Gong

**Affiliations:** 1 Department of Neurology, The First Affiliated Hospital of Anhui Medical University, Hefei, China; 2 Department of Neurology, Affiliated Yantai Yuhuangding Hospital of Qingdao University, Yantai, Shandong China; 3 Department of Cardiology, Affiliated Yantai Yuhuangding Hospital of Qingdao University, Yantai, Shandong China

**Keywords:** Astroglial autoimmunity, GFAP, Autonomic dysfunction, Heart rate variability, Blood pressure variability, Circadian rhythm, Case report

## Abstract

**Background:**

Autonomic dysfunctions including bladder dysfunction, gastrointestinal dysfunction and orthostasis are common symptoms of autoimmune glial fibrillary acidic protein astrocytopathy (A-GFAP-A); however, cardiac autonomic dysfunction and abnormal circadian rhythm of blood pressure, which can lead to poor prognosis and even sudden cardiac death, has never been reported in A-GFAP-A patient.

**Case presentation:**

A 68-year-old male Chinese patient presented to our hospital with headache, fever, progressive disturbance of consciousness, dysuria, and limb weakness. Abnormal heart rate variability and non-dipper circadian rhythm of blood pressure gradually developed during hospitalization, which is rare in A-GFAP-A. He had positive GFAP IgG in cerebrospinal fluid (CSF). Enhanced brian MRI showed uneven enhancement and T2 hyperintense lesions of medulla oblongata; Cervical spine MRI showed T2 hyperintense lesions in medulla oblongata and upper margin of the T2 vertebral body. A contrast-enhanced thoracic spine MRI showed uneven enhancement and T2 hyperintense lesions of T1 to T6 vertebral segments. After treatment with intravenous immunoglobulin and corticosteroids, the patient’s symptoms, including autonomic dysfunction, alleviated dramatically. Finally, his heart rate variability and blood pressure variability became normal.

**Conclusions:**

Our case broadens the spectrum of expected symptoms in A-GFAP- A syndromes as it presented with heart rate variability and blood pressure variability.

## Background

Autoimmune glial fibrillary acidic protein astrocytopathy (A-GFAP-A) is a newly discovered autoimmune nervous system disease involving the brain, spinal cord, meninges and optic nerve. Fang et al. [1] of the Mayo Clinic first reported and confirmed GFAP-IgG in cerebrospinal fluid (CSF) and/or serum as a specific biomarker for this disease in 2016. Clinically, A-GFAP-A often presents with headache, fever, encephalitis, myelitis, abnormal vision, tremor, dementia, and dysautonomia [2]. Approximately 20% of A-GFAP-A patients have autonomic dysfunction, mainly orthostasis, gastrointestinal dyskinesia, erectile dysfunction, bladder dysfunction [3]. However, cardiac autonomic dysfunction and abnormal circadian rhythm of blood pressure had rarely reported in A-GFAP-A.

Autoimmune encephalitis (AE) with dysautonomia, of course includes A-GFAP-A, may cause more morbidity and mortality [4], because autonomic dysfunction, especially cardiac autonomic dysfunction and abnormal circadian rhythm of blood pressure (CRBP), can complicate intensive care and involve a ventilator [5], even lead to hemodynamic shock sudden cardiac death (SCD). Therefore, we should pay more attention to cardiac autonomic dysfunction and abnormal CRBP in A-GFAP-A patients.

Heart rate variability (HRV) refers to changes in the heartbeat cycle [6], which mainly reflects a sensitive indicator of the dynamic balance state of cardiac autonomic nerve regulation [7–9] . It is widely used for assessing autonomic nervous function for decades, especially evaluating the balance between sympathetic and vagal nerves. Deceleration capacity of heart rate (DC) is an advanced marker of HRV and can quantitatively detect the function of vagal nerves [10]. As a method implementing the concept of DC, deceleration runs of heart rate (DRs) can quantify heart rate deceleration and vagal regulation of the sinoatrial node [11].

Herein, we report a rare case of A-GFAP-A resembling infectious encephalitis with obvious autonomic nervous disorder, especially including abnormity in HRV and CRBP as clinical manifestation, to broaden the spectrum of autonomic dysfunction types in A-GFAP-A.

## Case presentation

A 68-year-old male Chinese patient presented with headache and fever of up to 39 °C, accompanied by chills, paroxysmal cough and sputum, and systemic myalgia at the end of September 2021. The patient was treated with antiviral and antibacterial therapy that included moxifloxacin combined with piperacillin, tazobactam, and oseltamivir, and his body temperature declined and headache disappeared. The lumbar puncture (LP) revealed a opening pressure of 150 mmH_2_O, white blood count (WBC) of 149 × 10^6^/L (96.6% lymphocytes), decreased glucose level of 1.99 mmol/L, and significantly increased protein level of 2356.3 mg/dL. Initial brain magnetic resonance imaging (MRI) and electroencephalography were unremarkable. However, 1 week later, the patient gradually developed disturbance of consciousness with dysuria, and then was transferred to our hospital. The patient had the medical history of hypertension, and he worked as a farmer with frequent contact with pigs and sheep. Neurological examination revealed a lethargic state, stiff neck, weakened tendon reflex, positive Kernig’s sign, and positive bilateral pathological signs.

Differential diagnoses included inflection etiology (e.g., neurobrucellosis and tuberculous meningoencephalitis) and autoimmune etiology. As atypical bacterial meningitis or viral meningitis could not be ruled out, the patient received treatment with ceftriaxone sodium, rifampicin, doxycycline, and ganciclovir infusion. On admission, his serum inflammatory indicators were normal. Tests for antinuclear antibody, anti-neutrophil cytoplasmic antibody subtype, lupus anticoagulant, rheumatoid factor, autoantibody screen, anti-Hantavirus antibody (IgG, IgM), and Brucella antibody (IgG) were negative. Serum Epstein-Barr virus (EBV) DNA test indicated prior infection.

**Fig. 1:**
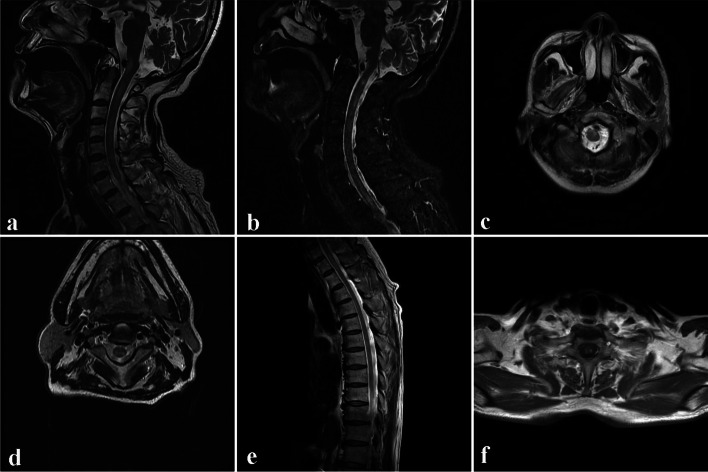
Magnetic resonance imaging of brain, cervical spine, and thoracic spine. Enhanced brian MRI showing uneven enhancement of medulla oblongata (**a**) and T2 hyperintensity lesions of medulla oblongata (**b**); Cervical spine MRI showing T2 hyperintense lesions in medulla oblongata and upper margin of the T2 vertebral body (**c**); A contrast-enhanced thoracic spine MRI showing uneven enhancement of T1 to T6 vertebral segments(**d**, **e**) and T2 hyperintense lesions of T1 to T6 vertebral segments (**f**)

**Fig. 2:**
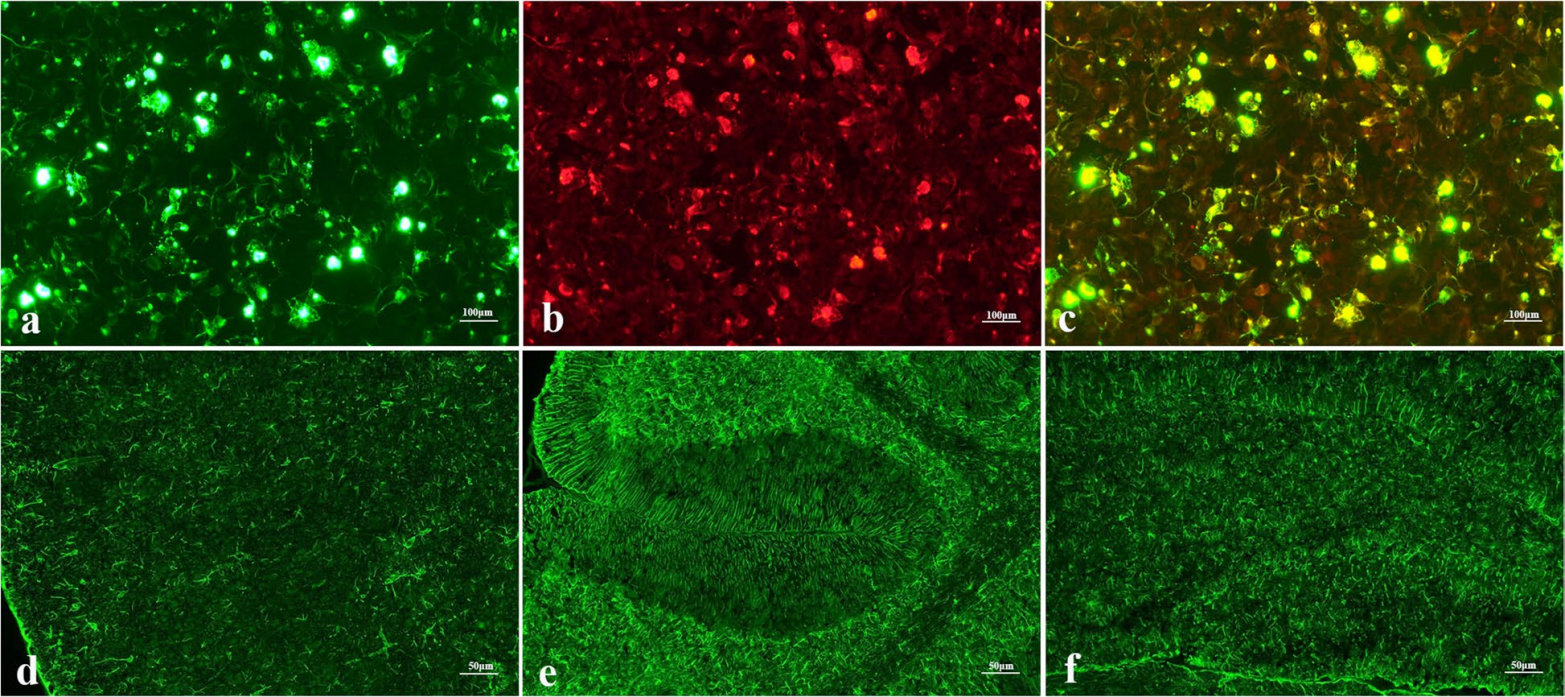
Cell-based assay of GFAP-transfected HEK293 cells and tissue-based immunofluorescence assay of CSF GFAP-IgG using rat brain. (**a**) HEK293 cells expressing green fluorescent protein (GFP)-labeled GFAP. (**b**) GFAP-IgG detected in CSF of patients; (**c**) Red fluorescence overlapping with specific sites of green fluorescence cells. Astrocyte fluorescence is widespread in the cortex (**d**) and hippocampus (**e**), and meningeal fluorescence is enhanced. Linear fluorescence is observed throughout the molecular layer in the cerebellum (**f**), and astrocyte fluorescence is observed in the granular layer and white matter

After 3 days of treatment, the patient’s consciousness gradually became clear and his neck stiffness improved, but he exhibited tremor in both upper limbs and weakened strength in both lower limbs, mainly in the right lower limb, which was accompanied by paresthesia. Enhanced brian MRI showed uneven enhancement and T2 hyperintense lesions of medulla oblongata (Fig. 1 a, b); Cervical spine MRI showed T2 hyperintense lesions in medulla oblongata and upper margin of the T2 vertebral body (Fig. 1 c). A contrast-enhanced thoracic spine MRI showed uneven enhancement and T2 hyperintense lesions of T1 to T6 vertebral segments (Fig. 1 d, e, f), reminiscent of autoimmune and demyelinating diseases. Electromyography was normal. LP showed a pressure of 80 mmH_2_0, WBC count of 76 × 10^6^/L (97.4% lymphocytes), normal glucose level (2.73 mmol/L), and protein level of 2356.3 mg/dL. Next-generation sequencing (NGS) of CSF showed 15 reads of EBV; Xpert, T-SPOT, and brucellosis antibody in the CSF and serum were negative. Autoimmune antibodies in the CSF and serum, including anti-N-methyl-D-aspartate receptor antibodies, anti-aquaporin 4, anti-myelin oligodendrocyte glycoprotein, and anti-contactin-associated protein-like 2 antibodies, anti-leucine-rich glioma-inactivated 1, anti-gamma-aminobutyric acid type receptor antibodies, anti-α amino-3-hydroxy-5-methyl-4-isoxazolepropionic acid 1/2 receptor antibodies were negative. However, GFAP-IgG in CSF was positive in both cell- and tissue-based assays (Fig. 2). The patient was diagnosed with A-GFAP-A, and then he was administered intravenous immunoglobulin (IVIg) and methylprednisolone.

**Fig. 3:**
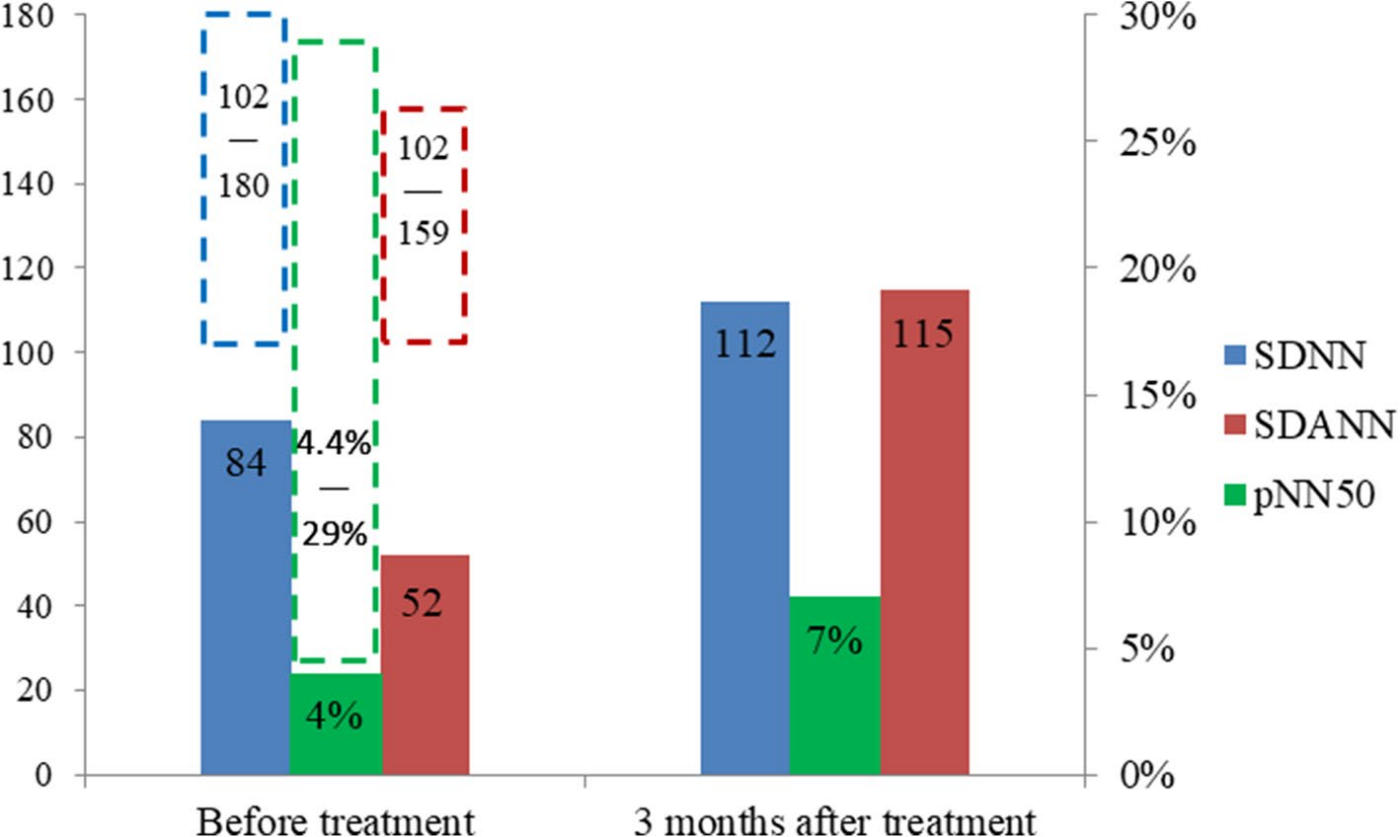
Abnormal indicators of heart rate variability before and 3 months after treatment. Before treatment, SDNN, SDANN and pNN50 decreased significantly, and 3 months after treatment, SDNN, SDANN and pNN50 increased to normal levels. (Dotted boxes indicate the normal range of each indicator: SDNN 102-180 ms, SDANN 102-159 ms, pNN50 4.4–29%)

The patient’s lower limb weakness and fever gradually improved, but developed dizziness, fatigue, and sweating after prolonged sitting (approximately 30 min). Ambulatory blood pressure monitoring showed a drop in systolic blood pressure of approximately 30 mmHg after 30 minutes of sitting, suggesting postural hypotension. The mean circadian rhythm variability in systolic blood pressure was non-dipper. 24-hour ambulatory electrocardiogram (Holter) showed a standard deviation of normal-to-normal interval (SDNN) of 84 ms, standard deviation of the average of normal-to-normal interval (SDANN) of 52 ms, and percentage of R-R interval difference > 50 ms (pNN50) of 4.4% (Fig. 3). Additionally, DRs suggested a medium risk of SCD (DR4 = 0.0649%, DR2 = 5.6803%, DR8 = 0.0024%). Electrocardiogram and echocardiography was normal. No abnormalities were observed on the thoracolumbar MRI.

After treatment with a standard IVIg regimen (0.4 g/kg body weight/day) for 5 days and pulses of 500 mg/d methylprednisolone for 3 days, 250 mg/d methylprednisolone for 3 days, 125 mg/d methylprednisolone for 3 days, and 60 mg/d prednisone with a dose reduction of 5 mg every 2 weeks, the patient’s fever disappeared and lower limb weakness improved. 3 months later, his orthostatic hypotension disappeared, SDNN, SDANN and pNN50 increased to normal levels (Fig. 3), blood pressure variability (BPV) became normal. Patient condition gradually improved and he could mobilise independently.

## Discussion

Clinically, the incidence of headache and fever in A-GFAP-A patients was 63.2 and 52.6%, respectively [12]. The initial clinical presentation of our case was fever, headache, and neck stiffness, accompanied by increased WBC count, decreased glucose, and increased CSF protein, making it easy to misdiagnose central nervous system infections, especially tuberculous meningoencephalitis. In addition, due to our patient’s history of exposure to pigs and sheep, neurobrucellosis was also considered. However, the results of comprehensive etiological examination (metagenomic NGS, Xpert, bacterial culture of CSF, and brucellosis antibody) were negative, there was minimal response to antimicrobial therapy, and extensive nervous system involvement appeared, suggesting the possibility of immune inflammation. At this moment, AE should be considered. To date, the diagnosis of A-GFAP-A mainly relies on the detection of GFAP antibodies in CSF or serum, which is not widely used in clinical practice. Clinicians face many challenges in the diagnosis of A-GFAP-A prior to GFAP-IgG testing. Therefore, when the etiology of patients suspected of having a central nervous system infection is negative, autoimmune inflammation should be considered when multiple parts of the nervous system are involved alongside empiric resistance to infection, GFAP-IgG should be tested to exclude A-GFAP-A.

Autonomic dysfunction is another common symptom of A-GFAP-A. For AE patients, accompanying with autonomic dysfunction are more in need of intensive care units hospitalization and mechanical ventilation, and is more likely associated with poor prognosis; The possible reason is dysregulation of blood pressure, arrhythmia and SCD [13]. By parity of reasoning, in patients with A-GFAP-A, autonomic dysfunction should be received more attention to obtain earlier therapy, improved quality of life and have lower mortality.

In our case, the most prominent symptoms were abnormal HRV and CRBP, which are rarely reported in A-GFAP-A. HRV, one of cardiac autonomic function indicator, is an important maker for predicting malignant arrhythmia and SCD. Its common data analysis index parameters include SDNN, SDANN, rMMD and pNN50 [14]; in addition, DRs, an advanced indicator of HRV, refers to the continuous occurrence of decelerating heart rate, which is the specific performance of sinus rhythm regulated by vagus nerve in a short period of time. In the present case, SDNN and SDANN decreased, suggesting enhanced sympathetic nerve activity, while the abnormal decrease in pNN50 suggested decreased vagal nerve activity; Abnormal DRs indicated that the vagal nerve excitability decreased, the protective effect on the body heart was weakened, and the risk of SCD increased in our patient. The mechanism of A-GFAP-A’s involvement in cardiac autonomic nervous function remains unclear. It has been reported that NMDA receptor antibodies may destroy the sympathetic circuits by controlling brainstem vagal nerve output and modulate sympathetic nerve output in hypothalamus and spinal cord [4], resulting in cardiac sympathetic dysfunction of anti-NMDAR encephalitis. A-GFAP-A is one type of autoimmune encephalitis, whether GFAP antibodies can attack sympathetic circuits through cytotoxic T cell-mediated autoimmune responses, thus affecting the stability of HRV, needs further research. Since GFAP can also be expressed in Schwann cells, peripheral nerves may also be a potential target for immune attack.

CRBP helps reveal circadian variations in blood pressure. According to the decrease of mean nocturnal systolic blood pressure compared with daytime (nocturnal drop rate), CRBP is divided into dipper (nocturnal drop rate ≥ 10%) and non-dipper (nocturnal drop rate < 10%). Dipper is thought to be a normal physiological condition. Non-dipper can be produced by autonomic dysfunction and is associated with target organ damage and an increased risk of cardiovascular death [15]. The CRBP of our case was non-dipper. The co-occurrence of abnormal CRBP and abnormal HPV further confirmed the existence of autonomic dysfunction. Weakened or disappeared CRBP can increase the risk of target organ damage and cardiovascular and cerebrovascular events. Early identification and treatment of abnormal HRV and BPV may improve the prognosis of A-GFAP-A.

In addition, our patient presented with urinary retention and orthostatic hypotension. It is unclear why A-GFAP-A has obvious phytophilic features. The limitation of this study was lack of sympathetic skin response test.

The etiology of A-GFAP-A is unknown, and approximately 30–40% of patients have tumors and infections [1, 2, 16]. In this study, the patient developed fever and headache, and NGS of CSF suggested EBV infection, but the patient’s serum results suggested that it might have been a previous infection. It is unclear whether A-GFAP-A in this patient was triggered by viral infection, and further clinical cases and studies are needed to confirm the correlation between EBV and A-GFAP-A. The possibility of other unknown microbial infections, potential tumors (no evidence of tumor was found in the examination of the common tumor in this case, and further follow-up is needed), or other causes for the immune response cannot be ruled out. The role of GFAP antibody as an antibody to intracellular antigen has not been unanimously recognized. It has been reported [17] that absence of astrocytes involvement was found in autopsies of GFAP-positive patients. GFAP encephalitis is sensitive to hormones, but the effect of human immunoglobulin on GFAP encephalitis is unclear and needs to be further studied in depth.

## Conclusion

Although A-GFAP-A has been newly discovered, it is gradually being recognized. When fever, encephalitis, myelitis, and autonomic dysfunction occur, A-GFAP-A should be considered. For patients with GFAP, HPV and ambulatory blood pressure monitoring should be detected, especially when they have vegetative neurological dysfunction such as dizziness and dysuria, to prevent the risk of SCD or fainting and falling. Conversely, when patients have unexplained autonomic neuropathy, GFAP antibody testing should be performed to confirm the diagnosis.

## Data Availability

Not applicable.

## Abbreviations

A-GFAP-A: Autoimmune glial fibrillary acidic protein astrocytoma
GFAP: Glial fibrillary acidic protein
CSF: Cerebrospinal fluid
HRV: Heart rate variability
SCD: Sudden cardiac death
CRBP: Circadian rhythm of blood pressure
LP: Lumbar puncture
WBC: White blood count
MRI: Magnetic resonance imaging
NGS: Next-generation sequencing
EBV: Epstein-Barr virus
IVIg: Intravenous immunoglobulin
SDNN: Standard deviation of normal-to-normal intervals
SDANN: Standard deviation of the average of normal-to-normal intervals
pNN50: Percentage of R-R interval difference > 50 ms
BPV: Blood pressure variability
AE: autoimmune encephalitis.
